# Macrophage PSRC1 attenuates atherosclerosis via extracellular vesicle-mediated MBD2 delivery to induce PCSK9 promoter methylation in hepatocytes

**DOI:** 10.1186/s13578-026-01598-9

**Published:** 2026-06-09

**Authors:** Tongwei Wu, Hui Huang, Xiaoqian Zhang, Xinyi Feng, Weiming Luo, Lilong Lin, Dan Liu, Hangyu Pan, Shaopeng Wang, Jierong Zhang, Xianyi Zhang, Zhigang Guo, Mengzhuo Yin, Shiping Cao, Yanan Zhang

**Affiliations:** 1 Department of Ultrasonics, Nanfang Hospital, Southern Medical University, Guangzhou, Guangdong China; 2 Department of Cardiology, State Key Laboratory of Organ Failure Research, Nanfang Hospital, Southern Medical University, 1838 North Guangzhou Avenue, Guangzhou, 510515 Guangdong China; 3 Cardiovascular Medicine Center, Heyou Hospital, No. 1 of Heren Road, Junlan Community, Beijiao Town, Shunde District, Foshan City, 528306 Guangdong China; 4 Department of Cardiology, Huiqiao Medical Center, Nanfang Hospital, Southern Medical University, Guangzhou, Guangdong China; 5 Department of Geriatrics, Guangzhou First People’s Hospital, School of Medicine, South China University of Technology, Guangzhou, Guangdong China; 6 Department of Cardiology, Guangzhou Development District Hospital, Guangzhou, Guangdong China; 7 College of Traditional Chinese Medicine, Xin Jiang Medical University, Xin Jiang, China

**Keywords:** PSRC1, Extracellular vesicles, Atherosclerosis

## Abstract

**Background:**

Atherosclerotic cardiovascular disease (ASCVD) is driven by dysregulated lipid metabolism and chronic inflammation. However, the mechanisms governing immune–liver crosstalk in this context remain poorly defined. Proline/serine-rich coiled-coil protein 1 (PSRC1) is a known regulator of cholesterol metabolism, but whether macrophage-derived PSRC1 influences hepatic functions via intercellular communication is unknown.

**Methods:**

The relationship between macrophage PSRC1 and hepatic PCSK9 was examined in patients with coronary artery disease and murine models. We employed AAV6-mediated macrophage-specific targeting and whole-body Psrc1⁻/⁻ mice to evaluate cell-type-specific effects. Macrophage–hepatocyte communication was investigated using transwell systems and genetic blockade of EV secretion (sh-Rab27a). The selectivity of EV cargo loading was validated by protease protection assays and TSG101 interaction analysis. In vivo EV tracking (DiI-labeling) and ChIP-qPCR for DNMT recruitment were performed to elucidate the systemic and epigenetic mechanisms.

**Results:**

Macrophage PSRC1 expression was significantly reduced in atherosclerotic conditions and inversely correlated with hepatic PCSK9 levels. Macrophage-specific PSRC1 depletion alone was sufficient to recapitulate the systemic hypercholesterolemia and accelerated atherosclerosis observed in whole-body knockout models. PSRC1 was found to interact with TSG101 to promote the selective loading of MBD2 into the EV lumen, a process confirmed by protease protection. These MBD2-enriched EVs were preferentially sequestered by the liver after systemic administration. Mechanistically, transferred MBD2 functioned as an epigenetic scaffold, recruiting DNA methyltransferases (DNMT1/3A) to the PCSK9 promoter to drive CpG hypermethylation and transcriptional repression. In vivo, administration of MBD2-enriched EVs significantly reduced hepatic PCSK9 protein, lowered plasma cholesterol, and enhanced plaque stability in ApoE⁻/⁻ mice.

**Conclusions:**

Our findings uncover a novel macrophage–liver epigenetic axis where macrophage PSRC1 controls systemic cholesterol homeostasis by regulating the EV-mediated delivery of MBD2. This “Reader-recruits-Writer” mechanism provides a refined understanding of immune–metabolic crosstalk and suggests that engineered EV-based MBD2 delivery represents a promising therapeutic strategy for ASCVD.

**Supplementary Information:**

The online version contains supplementary material available at 10.1186/s13578-026-01598-9.

## Introduction

Atherosclerotic cardiovascular disease (ASCVD) remains a leading cause of global mortality and morbidity [1]. The pathogenesis of atherosclerosis is closely linked to lipid accumulation, chronic inflammation, and oxidative stress [2, 3]. Macrophages play a central role in this pathological process by facilitating lipid uptake, modifying lipoproteins, and promoting cellular necrosis, thereby contributing to the formation of necrotic cores [4, 5]. Concurrently, the liver serves as a key regulator of systemic lipid metabolism and a site of hepatic inflammation [5]. Moreover, dysregulated lipid accumulation in hepatocytes can exacerbate systemic metabolic disturbances, a process that further drives atherogenesis [6]. Therefore, the “inflammatory-lipid axis”—comprising macrophage-mediated inflammatory responses and hepatocyte-regulated lipid metabolism—converges at the core of atherosclerotic initiation and progression.

Proline/serine-rich coiled-coil protein 1 (PSRC1), also known as DDA3, is a microtubule-associated protein initially recognized for its role in mitotic processes in eukaryotic cells [7]. Notably, through multi-omics and multi-trait analyses, PSRC1 has been prioritized as a high-confidence causal gene and a potential therapeutic target for dyslipidemia [8]. In our previous work, we provided compelling evidence that PSRC1 overexpression suppresses cholesteryl ester accumulation in foam cells and attenuates atherosclerosis by elevating plasma HDL-C levels and enhancing HDL functionality [9]. Conversely, PSRC1 deficiency promotes trimethylamine N-oxide (TMAO) production via modulation of gut microbiota and impairs cholesterol transport through activation of sulfotransferase 2B1b, thereby exacerbating atherosclerotic progression [10, 11]. Furthermore, we observed that overexpression of PSRC1 led to a decreasing trend in hepatic PCSK9 levels, though the precise molecular mechanism remains unclear [8, 9]. Given that PSRC1 is highly expressed in macrophages, it remains unclear how macrophage-specific PSRC1 influences hepatic lipid metabolism via intercellular communication—a mechanism warranting further investigation.

Inter-organ “crosstalk” is essential for maintaining systemic homeostasis, and macrophage-hepatocyte communication represents an emerging focus in metabolic disease research. Previous studies have demonstrated that macrophage-derived extracellular vesicles or specific cytokines, such as miRNAs, TNF-α and IL-6, significantly modulate hepatic lipid metabolism and inflammatory signaling [12–14]. In this study, we employed a co-culture system of macrophages and hepatocytes to investigate how macrophage-derived PSRC1 mediates the expression of PCSK9 in hepatocytes via extracellular vesicle delivery, thereby regulating lipid metabolism in hepatocytes. Elucidation of this macrophage–liver communication pathway not only deepens our understanding of the complex regulation of cholesterol metabolism but also unveils new potential therapeutic strategies for ASCVD by targeting PCSK9.

## Results

### Macrophage PSRC1 negatively correlates with hepatocyte PCSK9 expression in atherosclerosis

**Fig. 1:**
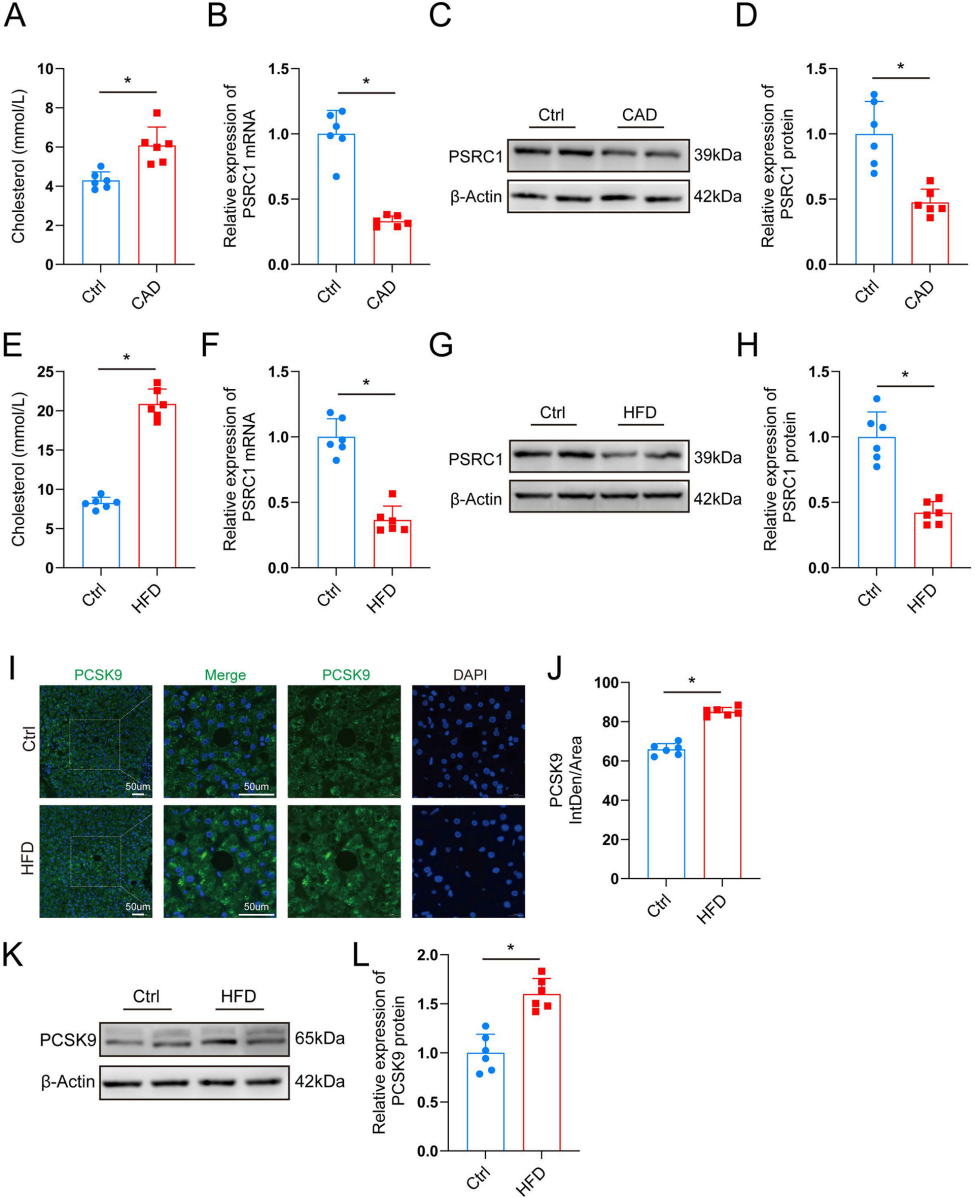
Macrophage PSRC1 negatively correlates with hepatocyte PCSK9 expression in atherosclerosis. **A** Comparison of serum cholesterol levels between patients with coronary artery disease (CAD) and non-CAD controls. **B** Relative mRNA expression of PSRC1 in peripheral blood monocyte-derived macrophages from CAD patients and non-CAD controls, as determined by qRT-PCR. **C**, **D** Representative immunoblots and quantitative analysis of PSRC1 protein expression in peripheral blood monocyte-derived macrophages from CAD patients and non-CAD controls. **E** Comparison of serum cholesterol levels between HFD–fed mice and normal diet–fed control mice. **F** Relative PSRC1 mRNA expression in peripheral blood monocyte-derived macrophages isolated from HFD-fed mice and control mice. **G**, **H** Representative immunoblots and densitometric analysis of PSRC1 protein expression in peripheral blood monocyte-derived macrophages from HFD-fed and control mice. **I**, **J** Representative immunofluorescence images and quantitative analysis of PCSK9 expression in liver sections from HFD-fed and control mice. Scale bar, 50 μm. **K**, **L** Representative immunoblots and quantitative analysis of PCSK9 protein expression in liver tissues from HFD-fed and control mice. *P < 0.05

To explore the relationship between macrophage PSRC1 and hepatic PCSK9, we first collected peripheral blood samples from a subset of patients with coronary artery disease (CAD) and healthy individuals without CAD, and isolated peripheral blood monocyte-derived macrophages. We found that serum cholesterol levels were significantly higher in CAD patients compared to healthy individuals without CAD (Fig. 1A). QRT-PCR assays showed that the mRNA expression of PSRC1 in peripheral blood monocyte-derived macrophages was significantly lower in CAD patients than in healthy individuals without CAD (Fig. 1B). Similarly, Western blot (WB) analysis revealed that the protein expression of PSRC1 in peripheral blood monocyte-derived macrophages was significantly lower in CAD patients than in healthy individuals without CAD (Fig. 1C, D).

Furthermore, we established a mouse atherosclerosis model through high-fat diet (HFD) feeding to further validate the above observations. We found that serum cholesterol levels were significantly higher in HFD-fed mice compared to normal diet-fed mice (Fig. 1E). QRT-PCR assays showed that the mRNA expression of PSRC1 in peripheral blood monocyte-derived macrophages was significantly lower in HFD-fed mice than in normal diet-fed mice (Fig. 1F). Similarly, Western blot analysis revealed that the protein expression of PSRC1 in peripheral blood monocyte-derived macrophages was significantly lower in HFD-fed mice than in normal diet-fed mice (Fig. 1G, H). Further immunofluorescence tissue staining experiments showed that the expression of hepatic PCSK9 was significantly higher in HFD-fed mice compared to normal diet-fed mice (Fig. 1I, J). Consistently, Western blot analysis also demonstrated that the protein expression of hepatic PCSK9 was significantly higher in HFD-fed mice than in normal diet-fed mice (Fig. 1K, L).

### Interfering with macrophage PSRC1 negatively regulates liver PCSK9 in arteriosclerosis

**Fig. 2:**
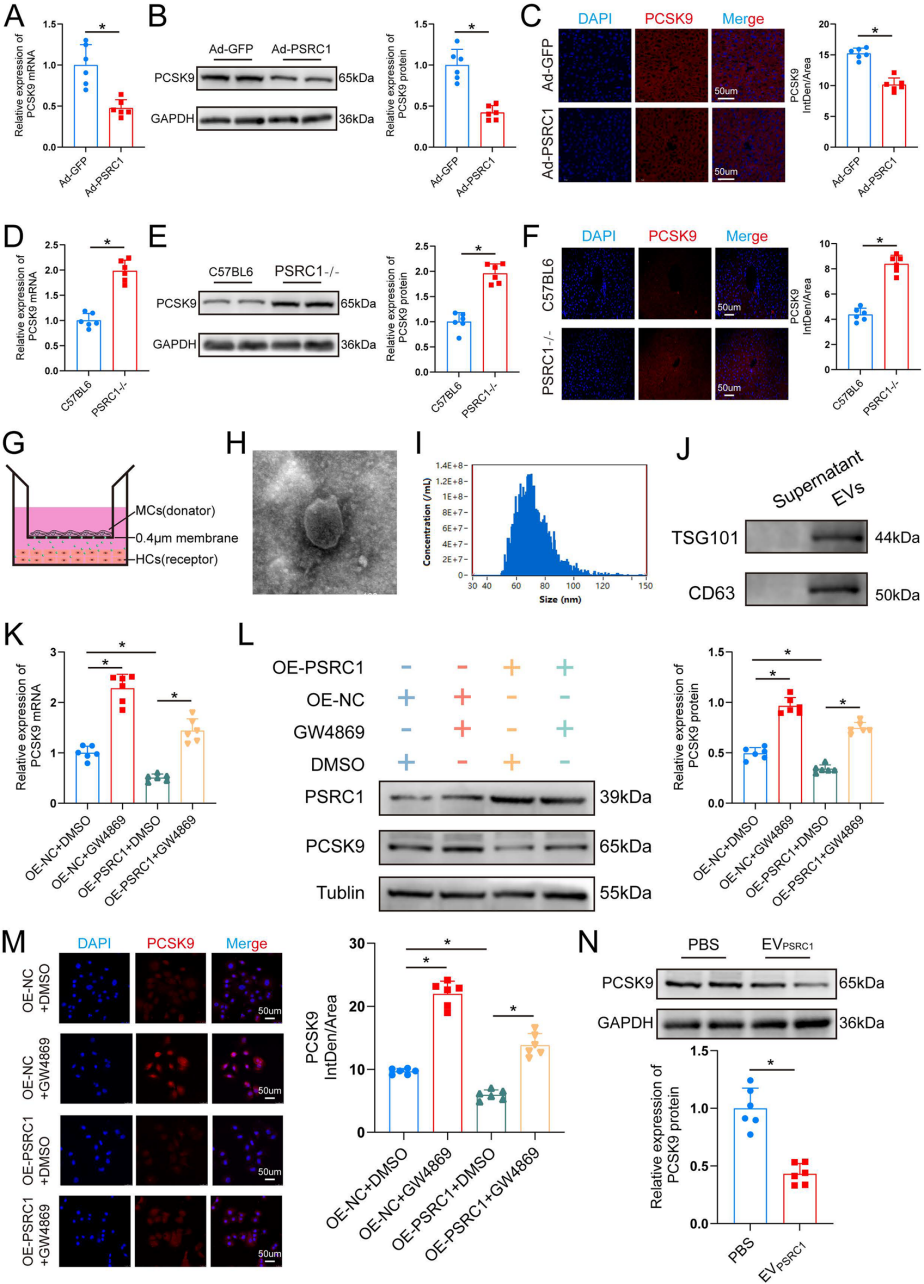
Interfering with macrophage PSRC1 negatively regulates liver PCSK9 in arteriosclerosis. **A** Relative PCSK9 mRNA expression in liver tissues from mice injected with adenovirus overexpressing PSRC1 (Ad-PSRC1) or control adenovirus (Ad-GFP). **B** Representative immunoblots and quantitative analysis of PCSK9 protein expression in liver tissues from Ad-PSRC1– and Ad-GFP–treated mice. **C** Representative immunofluorescence images and quantitative analysis of PCSK9 expression in liver sections from Ad-PSRC1– and Ad-GFP–treated mice. Scale bar, 50 μm. **D** Relative PCSK9 mRNA expression in liver tissues from PSRC1-deficient (PSRC1^−/−^) mice and C57BL/6 control mice. **E** Representative immunoblots and quantitative analysis of PCSK9 protein expression in liver tissues from PSRC1^^−/−^ and control mice. **F** Representative immunofluorescence images and quantitative analysis of PCSK9 expression in liver sections from PSRC1^^−/−^ and control mice. Scale bar, 50 μm. **G** Schematic illustration of the transwell-based macrophage–hepatocyte co-culture system. **H** Representative transmission electron microscopy images of EVs isolated from mouse macrophages. **I** Size distribution of macrophage-derived EVs as determined by nanoparticle tracking analysis (NTA). **J** Western blot characterization of EV markers in macrophage-derived EVs and corresponding culture supernatants. **K**–**M** In the macrophage–hepatocyte co-culture system, macrophages were transduced with Ad-PSRC1 and treated with the EV secretion inhibitor GW4869; PCSK9 mRNA expression **K**, protein levels by Western blot **L**, and immunofluorescence staining **M** were analyzed in hepatocytes. Scale bar, 50 μm. **N** EVs isolated from PSRC1-overexpressing macrophages were applied to hepatocytes, followed by analysis of PCSK9 expression. *P < 0.05

To further validate the regulatory relationship between macrophage PSRC1 and hepatic PCSK9 expression in vivo, we constructed PSRC1-overexpressing viral vector-transfected mice. Consistent with previous experiments, QRT-PCR assays showed that the mRNA expression of PCSK9 in the liver tissues of PSRC1-overexpressing mice was significantly lower than that in control mice (Fig. 2A). Western blot (WB) analysis similarly demonstrated that the protein expression of PCSK9 in the liver tissues of PSRC1-overexpressing mice was significantly lower than that in control mice (Fig. 2B). Further immunofluorescence tissue staining experiments revealed that the expression of PCSK9 in the liver tissues of PSRC1-overexpressing mice was significantly lower than that in control mice (Fig. 2C).

Additionally, we constructed whole-body PSRC1 knockout mice to further test the above hypothesis. We found that QRT-PCR assays showed the mRNA expression of PCSK9 in the liver tissues of PSRC1 knockout mice was significantly higher than that in control mice (Fig. 2D). Western blot analysis similarly demonstrated that the protein expression of PCSK9 in the liver tissues of PSRC1 knockout mice was significantly higher than that in control mice (Fig. 2E). Further immunofluorescence tissue staining experiments revealed that the expression of PCSK9 in the liver tissues of PSRC1 knockout mice was significantly higher than that in control mice (Fig. 2F).

To further corroborate whether the observed atherosclerotic phenotype is specifically driven by macrophage-derived PSRC1 rather than systemic or hepatocyte-intrinsic effects, we employed a macrophage-specific knockdown strategy using a recombinant adeno-associated virus serotype 6 (AAV6) vector. This vector carried shRNA targeting the mouse Psrc1 gene under the transcriptional control of the macrophage-specific F4/80 promoter (AAV6-F4/80-shPsrc1). Consistent with the findings in whole-body knockout mice, macrophage-specific silencing of PSRC1 significantly exacerbated atherosclerotic progression. Oil Red O staining of both the whole aorta and the aortic root sections revealed markedly increased plaque burden in the AAV6-F4/80-shPsrc1 group compared to the AAV-NC controls (Fig. S1A, D). Masson’s trichrome staining further demonstrated augmented fibrotic areas within the lesions of macrophage-PSRC1-deficient mice (Fig. S1B). Furthermore, immunohistochemical analysis showed an expansion of α-SMA-positive smooth muscle cell-rich regions (Fig. S1C), accompanied by a significant increase in CD68-positive macrophage infiltration within the aortic roots (Fig. S1E). These findings collectively substantiate that PSRC1 deficiency specifically in macrophages is a primary driver of atherosclerotic plaque instability and progression.

This raises the question of how macrophage PSRC1 affects the expression of PCSK9 in liver cells. Given the important role of extracellular vesicles (EVs) in intercellular communication, we hypothesized that macrophage PSRC1 acts on liver cells through EVs, thereby influencing PCSK9 expression in hepatocytes. To investigate this, we used a 0.4 μm pore diameter transwell system, where macrophages were seeded in the upper chamber and liver cells were cultured in the lower plate (Fig. 2G), to explore the effect of macrophage PSRC1 on hepatocyte PCSK9. Additionally, we extracted EVs from macrophages for further studies. The morphology, size distribution, and marker proteins of EVs were assessed by transmission electron microscopy (TEM), nanoparticle tracking analysis (NTA), and western blot analysis, respectively (Fig. 2H–J). To verify the regulatory effect of macrophage PSRC1 on hepatic PCSK9, we utilized a Transwell co-culture system. High-efficiency transfection of PSRC1 into macrophages was confirmed, with a transduction efficiency exceeding 90% (as shown in Fig. S2A). qRT-PCR and Western blot analysis demonstrated that PSRC1 overexpression in the upper chamber robustly suppressed PCSK9 expression in the recipient hepatocytes below. This inhibitory effect was significantly attenuated by the treatment with GW4869, an inhibitor of EV secretion (Fig. 2K, L). These findings were further corroborated by PCSK9 immunofluorescence in hepatocytes (Fig. 2M). More importantly, direct incubation of hepatocytes with isolated EVs from PSRC1-overexpressing macrophages consistently led to a substantial downregulation of PCSK9 protein levels (Fig. 2N), confirming that the macrophage-to-hepatocyte PSRC1/EV axis exert a potent regulatory effect on hepatic PCSK9 expression. To circumvent the potential off-target effects of GW4869 and definitively establish the dependency on the EV secretory pathway, we employed a genetic inhibition and rescue strategy. We first silenced Rab27a, a key GTPase required for exosome docking and secretion, in PSRC1-expressing macrophages. Genetic ablation of Rab27a significantly attenuated the ability of macrophages to suppress PCSK9 expression in recipient hepatocytes, leading to a marked increase in PCSK9 protein levels (Fig. S3A). Notably, this inhibitory effect was fully restored when hepatocytes were supplemented with isolated, macrophage-derived EVs. These ‘loss-of-secretion and rescue’ findings substantiate that the PSRC1-driven negative regulation of hepatic PCSK9 is specifically mediated via EV-dependent intercellular communication rather than non-vesicular soluble factors. These results indicate that macrophage PSRC1 negatively regulates PCSK9 expression in liver cells, and this intercellular communication is mediated, at least in part, through extracellular vesicles.

### Macrophage PSRC1 binds to TSG101 to promote the entry of MBD2 into EVs

**Fig. 3:**
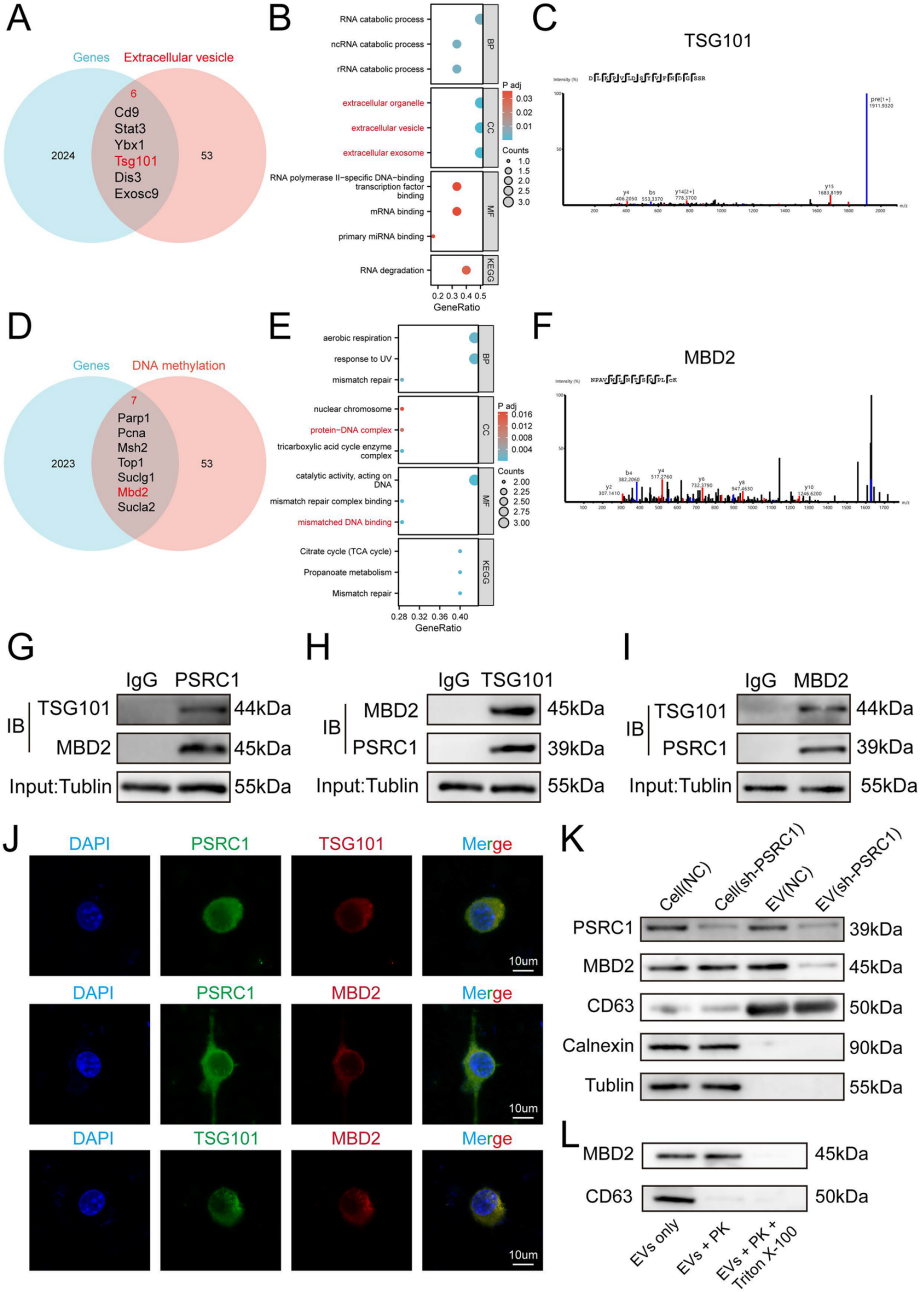
Macrophage PSRC1 binds to TSG101 to promote the entry of MBD2 into EVs. **A** Venn diagram showing the overlap between proteins identified by PSRC1 pulldown assays (n = 2030) and the top 60 genes associated with extracellular vesicle biogenesis. **B** Gene Ontology (GO) enrichment and KEGG pathway analyses of the six overlapping candidate genes. **C** Mass spectrometry peak map identifying TSG101 protein. **D** Venn diagram showing the overlap between PSRC1 pulldown–identified proteins and the top 60 genes associated with DNA methylation. **E** GO enrichment and KEGG pathway analyses of the seven overlapping candidate genes. **F** Mass spectrometry peak map identifying MBD2 protein. **G**–**I** Co-immunoprecipitation (co-IP) assays demonstrating reciprocal interactions among PSRC1, TSG101, and MBD2. **J** Immunofluorescence staining showing pairwise colocalization of PSRC1 with TSG101 and MBD2 in macrophages. Scale bar, 50 μm. **K** Representative Western blot analysis of PSRC1 and MBD2 expression in macrophage cell lysates and their secreted extracellular vesicles. Macrophages were transfected with scramble siRNA (NC) or PSRC1-specific shRNA (sh-PSRC1). Tubulin was used as the loading control for cell lysates. CD63 served as positive markers for EVs. Calnexin was included as a negative marker to verify the purity of the isolated EV fractions and the absence of cellular contamination. **L** Representative Western blot of a protease protection assay performed on isolated macrophage-derived EVs. EV fractions were treated with Proteinase K (PK, 50 ug/mL) in the presence or absence of 1% Triton X-100 (TX-100) for 30 min at 37 ℃. MBD2 remained intact after PK treatment but was degraded upon membrane disruption with TX-100. CD63 served as a positive control for EV integrity, showing characteristic depletion patterns upon detergent treatment. *P < 0.05

To identify the specific molecular mediators through which macrophage PSRC1 regulates hepatic PCSK9 via EVs, we performed a multi-step filtration strategy. Mass spectrometry analysis of PSRC1-associated proteins identified 2030 candidates. By intersecting these with the top 60 genes associated with EV biogenesis in public databases, we identified six candidate proteins: Cd9, Stat3, Ybx1, Tsg101, Dis3, and Exosc9 (Fig. 3A). GO and KEGG analyses confirmed their enrichment in vesicular transport pathways (Fig. 3B). Among these, Tsg101—a core component of the ESCRT-I complex—was prioritized for further investigation due to its well-established role in sorting protein cargo into exosomes. Parallelly, to identify epigenetic mediators, the 2030 proteins were intersected with the top 60 DNA methylation-related genes, yielding seven candidates: Parp1, Pcna, Msh2, Top1, Suclg1, Mbd2, and Sucla2 (Fig. 3D). Given our observation of Pcsk9 promoter hypermethylation, we focused on Mbd2, a critical ‘reader’ that bridges DNA methylation to transcriptional silencing. Preliminary Co-IP screening and literature-based functional scoring indicated that Tsg101 and Mbd2 showed the strongest physical and functional association with PSRC1 in the context of lipid-related signaling, leading us to select them as the primary targets for subsequent validation (Fig. 3G–I). Consistent with these observations, immunofluorescence colocalization analyses demonstrated pronounced spatial overlap of PSRC1 with both TSG101 and MBD2 within macrophages (Fig. 3J), further supporting the formation of a PSRC1–TSG101–MBD2 protein complex. To determine whether PSRC1 is essential for the selective sorting of MBD2 into macrophage-derived EVs, we performed loss-of-function experiments using PSRC1-specific siRNA. While Western blot analysis indicated that PSRC1 knockdown did not alter the endogenous expression of MBD2 within the macrophages themselves, it resulted in a dramatic reduction of MBD2 levels within the isolated EVs (Fig. 3K). These data provide critical evidence that PSRC1 is not merely an accessory protein, but a requisite mediator for the efficient packaging of MBD2 into the EV compartment, distinguishing this targeted loading mechanism from independent or stochastic protein secretion. To further delineate the sub-vesicular localization of MBD2 and exclude the possibility of non-specific external association or co-precipitation during isolation, a protease protection assay was performed on purified macrophage-derived EVs. Western blot analysis revealed that MBD2 remained protected from degradation upon treatment with Proteinase K alone. However, the addition of the detergent Triton X-100, which disrupts the EV lipid bilayer, rendered MBD2 susceptible to complete enzymatic digestion (Fig. 3L). These results definitively demonstrate that MBD2 is sequestered within the internal lumen of the EVs rather than being externally attached to the vesicle surface. Collectively, our data suggest that PSRC1 acts as a pivotal mediator that facilitates the active and selective packaging of the MBD2-TSG101 complex into the EV compartment, thereby protecting the cargo during intercellular transport.

### Macrophage EV MBD2 inhibits atherosclerosis progression in vivo

**Fig. 4:**
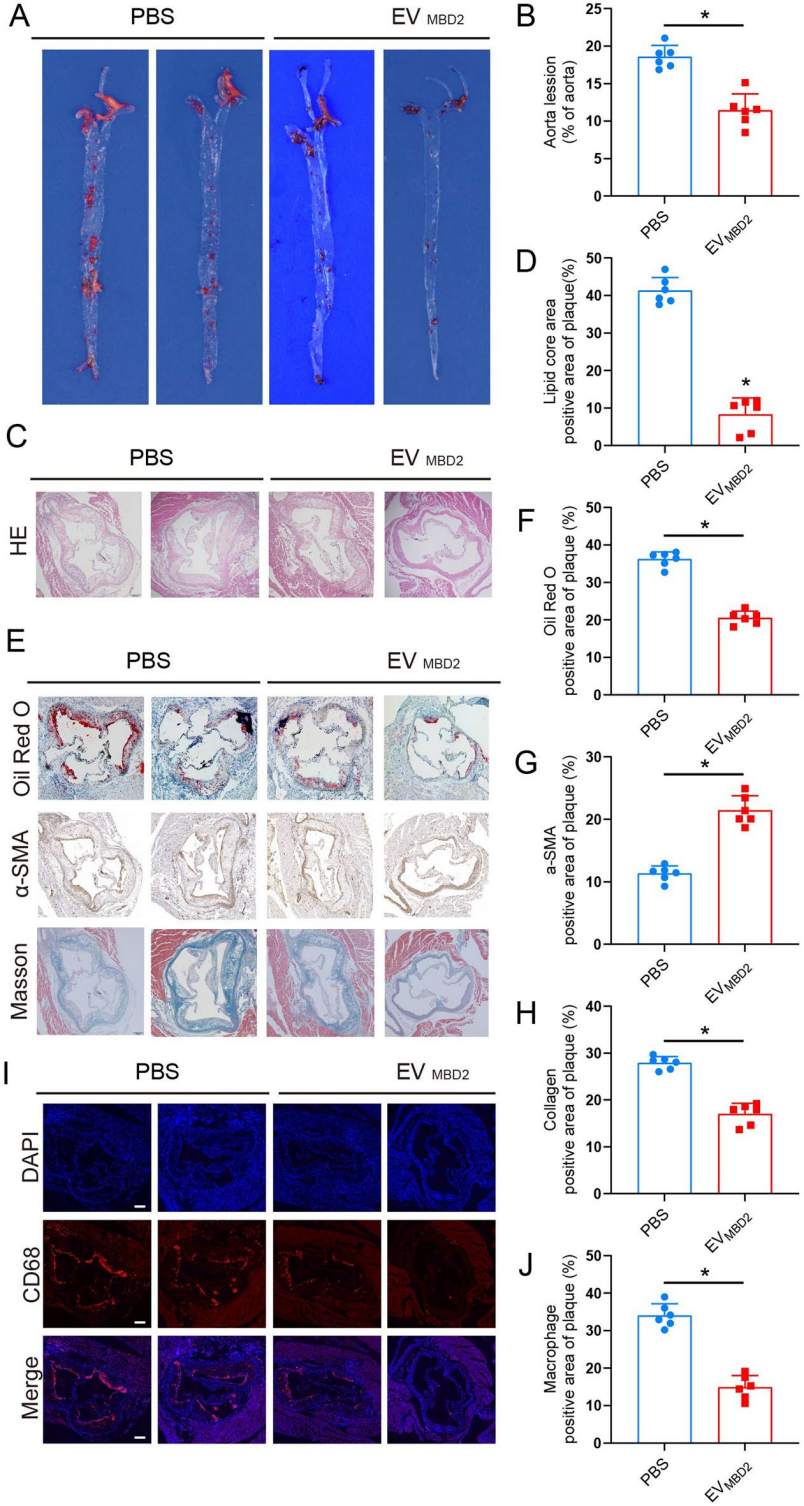
Macrophage EV MBD2 inhibits atherosclerosis progression in vivo. **A** ApoE^−/−^ mice were intravenously injected via the tail vein with EVs derived from MBD2-overexpressing macrophages (EV_MBD2_) or phosphate-buffered saline (PBS), followed by HFD feeding to induce atherosclerosis. Aortic tissues were subsequently harvested for analysis. **A**, **B** Representative en face Oil Red O staining of aortic plaques. **C**, **D** Hematoxylin and eosin (H&E) staining of aortic root sections showing lipid core areas. Scale bar, 200 μm. **E**–**J** Cross-sections of aortic roots stained with Oil Red O for lipids, α-smooth muscle actin (α-SMA) for smooth muscle cells, Masson’s trichrome for collagen deposition, and CD68 for macrophage infiltration. Scale bar, 200 μm. *P < 0.05

To investigate the impact of MBD2 on the progression of atherosclerosis, we first constructed an adenoviral vector (Adv-MBD2) to induce MBD2 overexpression in macrophages. EVs were subsequently isolated from the macrophage culture supernatant and intravenously administered to ApoE^−/−^ mice via the tail vein. To confirm the hepatic targeting and functional impact of administered EVs in vivo, we first evaluated the systemic biodistribution of DiI-labeled EV-MBD2. Live animal fluorescence imaging at 12 h post-tail vein injection revealed a predominant accumulation of the labeled EVs within the liver (Fig. S4A). To further establish the link between EV delivery and hepatic PCSK9 modulation, we assessed PCSK9 protein levels in liver tissues. Consistent with our in vitro findings, systemic administration of EV-MBD2 significantly suppressed hepatic PCSK9 protein expression compared to the PBS-treated control group (Fig. S4B). These results provide direct evidence that macrophage-derived EVs are efficiently sequestered by the liver and exert a potent inhibitory effect on hepatic PCSK9 in vivo, supporting the hepatic mechanism as a primary driver of the observed atheroprotective effects. Macroscopic Oil Red O staining of the aorta revealed a marked reduction in plaque burden in mice treated with EV-MBD2 compared with control animals (Fig. 4A, B). Consistently, H&E staining of the aortic root demonstrated a significant decrease in lesion area in the EV-MBD2 group (Fig. 4C, D). These findings indicate that EVs enriched in MBD2 effectively attenuate atherosclerotic plaque development.

Further assessment of plaque composition suggested an enhancement of plaque stability. Oil Red O and Masson’s trichrome staining revealed reduced triglyceride deposition and increased fibrotic content in the aortic roots of EV-MBD2–treated mice (Fig. 4E–H). α-SMA staining showed an expansion of smooth muscle cell (SMC)–rich areas (Fig. 4E, G), whereas CD68 immunohistochemistry demonstrated diminished macrophage infiltration (Fig. 4I, J). Collectively, these results suggest that MBD2-containing EVs not only reduce plaque size but also enhance aortic root plaque stability.

### Knockdown of MBD2 promotes atherosclerosis progression in vivo

**Fig. 5:**
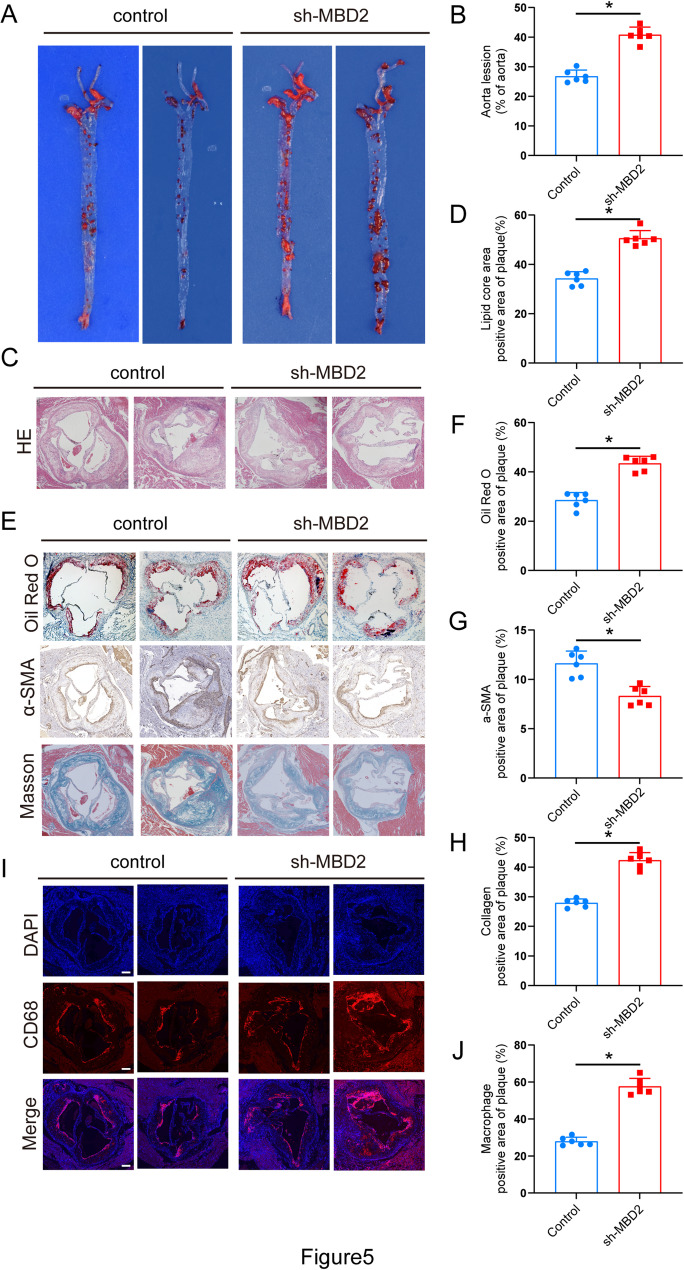
Knockdown of MBD2 promotes atherosclerosis progression in vivo. **A** ApoE^−/−^ mice were intravenously injected via the tail vein with adenovirus encoding shRNA targeting MBD2 (sh-MBD2) or saline, followed by HFD feeding to establish an atherosclerosis model. Aortic tissues were collected for further analysis. **A**, **B** Representative en face Oil Red O staining of aortic plaques. **C**, **D** H&E staining of aortic root sections showing lipid core areas. Scale bar, 200 μm. **E**–**J** Cross-sections of aortic roots stained with Oil Red O, α-SMA, Masson’s trichrome, and CD68 to evaluate lipid accumulation, smooth muscle cell content, collagen deposition, and macrophage infiltration, respectively. Scale bar, 200 μm. *P < 0.05

To validate the functional significance of MBD2 during atherosclerosis progression, an adenoviral vector expressing shRNA against MBD2 (Ad-shMBD2) was constructed and delivered to ApoE^−/−^ mice via tail-vein injection. Oil Red O staining of whole aortas and aortic roots revealed significantly larger plaques in the sh-MBD2 group relative to controls (Fig. 5A, B). H&E and Masson staining further showed increased fibrotic areas within the aortic root lesions of sh-MBD2–treated mice (Fig. 5C–E, H). Cross-sectional Oil Red O staining demonstrated an enlarged lipid core following MBD2 knockdown (Fig. 5E, F). Immunohistochemical staining for α-SMA indicated an expansion of SMC-rich regions (Fig. 5E, G), whereas CD68 immunofluorescence revealed enhanced macrophage infiltration in aortic roots of MBD2-deficient mice (Fig. 5I, J). Taken together, these findings suggest that loss of MBD2 significantly reduces plaque stability and accelerates atherosclerosis progression.

### MBD2 regulates PCSK9 transcription through CpG methylation–dependent binding

To definitively track the intracellular trafficking of EV-shuttled MBD2 and distinguish it from the endogenous pool in recipient hepatocytes, we utilized EVs isolated from macrophages overexpressing MBD2 (Flag-MBD2). Hepatocytes were incubated with these Flag-MBD2-containing EVs and monitored via confocal immunofluorescence at different time intervals. At 2 h post-incubation, the exogenous Flag-MBD2 signal was primarily detected within the cytoplasm of hepatocytes. By 24 h, a significant proportion of the Flag-tagged protein had translocated into the nucleus, co-localizing with DAPI staining (Fig. S5A). This time-dependent shift from the cytoplasm to the nucleus provides direct visual evidence that EV-delivered MBD2 successfully crosses the plasma membrane and subsequently undergoes nuclear translocation, reaching its functional site to regulate host gene transcription.

**Fig. 6:**
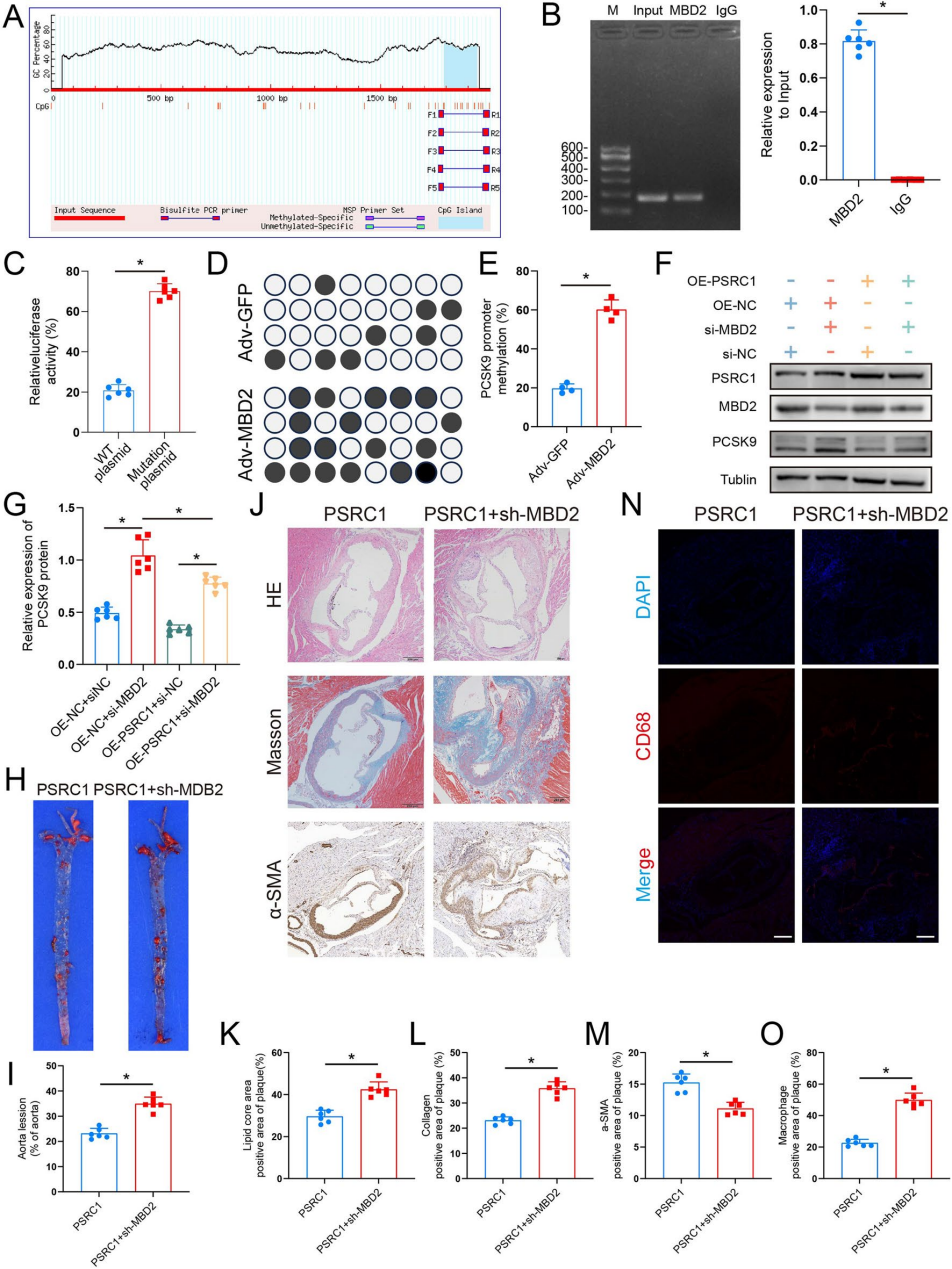
MBD2 regulates PCSK9 transcription through CpG methylation–dependent binding. **A** Predicted CpG island distribution within the PCSK9 promoter and the locations of five primer pairs generated using MethPrimer 2.0. **B** ChIP-PCR analysis demonstrating the binding of MBD2 to the PCSK9 promoter region. **C** Relative luciferase activity in hepatocytes transfected with wild-type or methyl-CpG binding–deficient MBD2 plasmids. **D**, **E** Bisulfite sequencing PCR (BSP) analysis of selected CpG sites within the PCSK9 promoter and quantitative assessment of methylation levels. **F**, **G** In macrophage–hepatocyte co-culture systems, rescue experiments showing the effects of macrophage PSRC1 overexpression combined with MBD2 knockdown on hepatocyte PCSK9 protein expression. **H**–**O** In vivo rescue experiments in atherosclerotic mouse models demonstrating the effects of PSRC1 overexpression combined with MBD2 knockdown, as assessed by Oil Red O, Masson’s trichrome, H&E, α-SMA, and CD68 staining. Scale bar, 200 μm. *P < 0.05

Extensive previous research has demonstrated that MBD2 regulates gene expression by binding to methylated CpG island-enriched regions within gene promoters and modulating their methylation status [15–17]. To explore how MBD2 modulates PCSK9 expression, MethPrimer Promoter 2.0 was used to predict CpG islands within the PCSK9 promoter, and corresponding primers were designed(Fig. 6A) [18]. We next determined whether MBD2 selectively binds hypermethylated CpG sites within this region. Chromatin immunoprecipitation (ChIP) analysis confirmed that MBD2 interacts with the PCSK9 promoter between − 1943 and − 1790 bp, a region containing hypermethylated CpG motifs (Fig. 6A, B). Luciferase reporter assays demonstrated that hepatocytes transfected with a CpG-deficient mutant promoter construct exhibited significantly higher PCSK9 transcriptional activity compared with those transfected with the wild-type promoter (Fig. 6C), indicating that CpG methylation is required for MBD2-dependent repression. Furthermore, bisulfite sequencing revealed a substantially higher proportion of methylated CpG sites in the PCSK9 promoter in MBD2-overexpressing samples compared with controls (Fig. 6D, E). Given that MBD2 is primarily a methyl-CpG binding protein rather than a de novo methyltransferase, we sought to elucidate the precise mechanism by which it elevates PCSK9 methylation. We hypothesized that MBD2 might function as a molecular scaffold to recruit DNA methyltransferases (DNMTs) to the PCSK9 locus. Co-immunoprecipitation (Co-IP) assays in hepatocytes confirmed a physical interaction between MBD2 and both DNMT1 and DNMT3A (Fig. S6A). To further determine whether MBD2 mediates the genomic recruitment of these enzymes, we performed Chromatin Immunoprecipitation (ChIP)-qPCR. The results revealed that overexpression of MBD2 (OE-MBD2) significantly enhanced the enrichment of both DNMT1 and DNMT3A at the PCSK9 promoter region compared to the OE-NC group (Fig. S6B, C). In contrast, the enrichment in the IgG control groups remained negligible. These findings suggest that EV-delivered MBD2 suppresses PCSK9 transcription by acting as a bridging protein that recruits DNMT1 and DNMT3A to the PCSK9 promoter, thereby facilitating the maintenance and spread of DNA methylation.

Then, rescue experiments were performed to validate the PSRC1/MBD2/PCSK9 regulatory axis both in vitro and in vivo. Western blot analysis demonstrated that PSRC1 overexpression markedly decreased PCSK9 protein levels, an effect reversed by MBD2 inhibition (Fig. 6F, G). Consistently, in vivo staining assays—including Oil Red O, Masson, H&E, α-SMA, and CD68—showed that the atheroprotective effects of PSRC1 were largely abolished upon MBD2 silencing (Fig. 6H–O). Taken together, these findings demonstrate that MBD2-containing EVs interact with methylated CpG motifs in the PCSK9 promoter, thereby mediating its transcriptional repression and contributing to the attenuation of atherosclerosis progression.

## Discussion

**Fig. 7:**
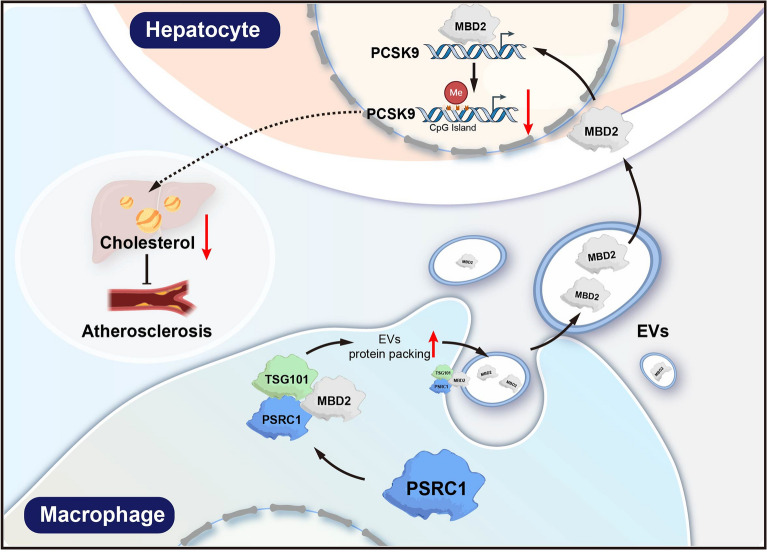
Graphical abstract of the study. Circulating macrophage-derived PSRC1 binds to TSG101 and MBD2, thereby facilitating the loading of MBD2 into EVs for delivery to hepatocytes. Subsequently, MBD2 binds to CpG island regions within the PCSK9 promoter in hepatocytes, modulating its methylation status. This leads to transcriptional silencing of PCSK9, which regulates hepatocyte lipid metabolism and ultimately attenuates the progression of atherosclerosis

In this study, we reveal a previously unappreciated inter-organ regulatory circuit in which circulating macrophages exert a substantial influence on hepatic lipid metabolism, thereby modulating systemic cholesterol homeostasis and the progression of atherosclerosis (Fig. 7). While macrophages have traditionally been viewed as local effector cells within atherosclerotic lesions—engaging in lipid uptake, inflammatory activation, and foam cell formation—our findings extend their biological impact to remote metabolic organs. Specifically, we demonstrate that macrophage-derived signals can modulate hepatocyte PCSK9 expression and downstream lipid metabolic pathways, revealing macrophages as a critical upstream regulator in the inflammatory–lipid axis that drives atherosclerosis.

A major finding of our work is that macrophage-derived EVs serve as an essential communication channel facilitating this regulatory process. Although EVs have been implicated in macrophage-mediated inflammatory responses and crosstalk with endothelial cells or smooth muscle cells, their role in modulating hepatocyte cholesterol metabolism remains largely unexplored [19–21]. Using a transwell co-culture system, macrophage EV isolation, pharmacological inhibition, and gain-/loss-of-function models, we demonstrate that macrophage PSRC1 suppresses PCSK9 expression in hepatocytes in an EV-dependent manner. To overcome the potential off-target effects of pharmacological inhibitors like GW4869, we further employed a genetic approach using Rab27a silencing to block EV secretion. The subsequent rescue of hepatic Pcsk9 expression by adding back isolated EVs provides definitive evidence that the regulatory signal is physically encapsulated within the EVs rather than being a non-vesicular soluble factor. These observations strongly support that at least part of the metabolic influence exerted by macrophages on hepatocytes is mediated by EV cargo trafficking rather than soluble cytokines alone. This conceptual framework expands the understanding of how innate immune cells participate in systemic metabolic regulation.

One of the most important mechanistic insights from this study is the identification of MBD2 as a key messenger molecule within macrophage EVs. MBD2 is a canonical DNA methylation “reader” and an important epigenetic regulator known to mediate gene repression in immune responses, tumor progression, and metabolic disorders [22–24]. However, no previous study has demonstrated that MBD2 can be selectively packaged into EVs and transferred between organs to modulate gene expression in a distant tissue. Our findings provide the first evidence that macrophage PSRC1 physically interacts with TSG101—a central component of the ESCRT machinery—to promote the incorporation of MBD2 into EVs. This PSRC1–TSG101 interaction establishes a selective loading mechanism rather than a passive EV cargo effect. This targeted sorting process was further validated by a protease protection assay, which confirmed that MBD2 is sequestered within the internal lumen of the EVs, shielding it from extracellular proteolysis. Furthermore, we elucidated that MBD2, as a "reader" protein, actively recruits DNA methyltransferases (DNMT1 and DNMT3A) to the Pcsk9 promoter upon its entry into hepatocytes. This "Reader-recruits-Writer" model clarifies how an epigenetic reader can drive de novo methylation at a specific genomic locus, thereby ensuring stable transcriptional repression.

Upon entry into hepatocytes, the transferred MBD2 binds directly to methylated CpG regions within the PCSK9 promoter, resulting in transcriptional repression. Since PCSK9 is a master regulator of LDL receptor degradation and plasma LDL-C levels, the macrophage PSRC1–EV–MBD2 pathway represents a previously unknown epigenetic axis by which immune cells influence systemic cholesterol handling. This epigenetic mode of inter-organ regulation may extend beyond PCSK9 and could represent a generalizable mechanism for macrophage-mediated metabolic control.

Our in vivo experiments further confirm the biological significance of this pathway. By utilizing a dual-specificity approach—AAV6 serotype combined with a macrophage-specific F4/80 promoter—we successfully achieved macrophage-targeted Psrc1 knockdown. This targeted genetic manipulation recapitulated the pro-atherogenic phenotype of the whole-body knockout model, effectively isolating the macrophage-specific contribution from hepatocyte-intrinsic effects. Additionally, in vivo bioluminescence imaging of DiI-labeled EVs confirmed their preferential accumulation in the liver, where they successfully suppressed PCSK9 protein levels. These findings position MBD2 as a novel atheroprotective factor acting through repression of PCSK9. Importantly, they also highlight the potential of engineering EV-based delivery systems to target hepatic cholesterol metabolism, providing an innovative therapeutic alternative to conventional PCSK9 inhibition.

Despite these important findings, our study has several limitations. First, although we employed AAV-mediated macrophage-specific targeting to complement our whole-body PSRC1 knockout mice, future studies using stable Psrc1fl/fl; LysM-Cre mouse lines would provide even more permanent genetic evidence for these macrophage-specific effects. Second, the clinical data analyzed in this study were restricted to serum samples and monocyte-derived macrophages from CAD patients. Larger clinical cohorts, inclusion of liver tissue analysis, or longitudinal follow-up would increase the translational robustness of our observations. Third, macrophage EVs may carry additional proteins, non-coding RNAs, or chromatin-associated factors that might synergize with MBD2 or independently influence hepatocyte lipid metabolism. High-resolution EV proteomic and transcriptomic profiling will be necessary to uncover the broader landscape of macrophage EV-mediated epigenetic regulation. Furthermore, the upstream regulatory signals that modulate PSRC1 expression in macrophages remain unclear. It will be important to determine whether PSRC1 is responsive to dietary lipids, inflammatory stimuli, metabolic stressors, or plaque microenvironmental signals. Understanding these upstream cues will help integrate the PSRC1–MBD2–PCSK9 axis into broader models of immune–metabolic communication.

In summary, our study uncovers a novel inter-organ communication mechanism in which macrophage PSRC1 controls hepatic PCSK9 expression by regulating the EV-dependent transfer of MBD2 to hepatocytes. This macrophage–liver epigenetic axis offers mechanistic insight into how inflammatory cells exert long-range control over systemic cholesterol metabolism and atherosclerosis progression. The identification of MBD2 as a transferable epigenetic repressor also provides new therapeutic opportunities, including macrophage-targeted modulation of PSRC1, engineered EV-based MBD2 delivery, or combined approaches with existing lipid-lowering therapies. Collectively, our findings broaden the understanding of immune–metabolic interactions and open new avenues for the prevention and treatment of ASCVD.

## Materials and methods

### Ethics

The present study was approved by the Ethics Committee of Nanfang Hospital, Southern Medical University (NFEC‐2017‐K3‐Revision02) and was conducted in accordance with the principles of the Declaration of Helsinki. Written informed consent was obtained from all participants. All animal procedures were approved by the Animal Ethics Committee of Nanfang Hospital, Southern Medical University (IACUC-PROJECT-20251113-001). Experiments were conducted in accordance with the National Institutes of Health Guide for the Care and Use of Laboratory Animals.

### Animal model

ApoE^−/−^ mice on a C57BL/6 J background were purchased from Viewsolid Biotech Co., Ltd. (Beijing, China). PSRC1^−/−^ mice were generated from ApoE^−/−^ mice using the CRISPR–Cas9 system to delete exon 4 of the PSRC1 gene by the same company. Atherosclerosis (AS) model: Mice were fed a chow diet until 8 weeks of age, followed by a HFD for 12 weeks.

### Isolation of human monocytes

Human peripheral blood mononuclear cells (PBMCs) were isolated from whole blood using density gradient centrifugation with Ficoll-Paque Plus (BD Biosciences). Monocytes were subsequently purified from PBMCs by plastic adherence. The isolated monocytes were cultured in RPMI 1640 medium supplemented with 10% FBS at 37 °C in a humidified atmosphere of 5% CO_2_.

### Mouse peritoneal macrophage isolation

First, euthanize the mouse and perform an intraperitoneal lavage by injecting 5–8 mL of ice-cold PBS, followed by gentle abdominal massage and fluid aspiration. Then, centrifuge the collected lavage fluid at 300 × g for 5 min at 4 °C. Following this, resuspend the cell pellet in complete culture medium and plate the cells. After a 2–4 h adherence period, gently wash the plate to remove non-adherent cells; the remaining adherent population consists of primary peritoneal macrophages.

### Cell and tissue immunofluorescence

Immunostaining was performed on cultured cells and frozen tissue sections according to established protocols with modifications [25]. Briefly, samples were fixed with 4% paraformaldehyde for 15 min at room temperature, permeabilized using 0.3% Triton X-100 for 10 min, and then blocked with 5% bovine serum albumin (BSA) for 1 h to minimize nonspecific antibody binding. Primary antibody incubation was performed overnight at 4 °C using antibodies against CD68 (ab213363, Abcam), PCSK9 (ab185194, Abcam), MBD2 (sc-514062, Santa Cruz), TSG101 (ab320807, Abcam), and PSRC1 (bs-7619R, Bioss), all diluted in blocking buffer. Following extensive washing, samples were incubated for 1 h at room temperature in the dark with appropriate fluorochrome-conjugated secondary antibodies. Cell nuclei were counterstained with DAPI. Finally, coverslips (cells) or tissue sections were mounted using an antifade mounting medium. Images were acquired using a confocal fluorescence microscope.

### Western blot analysis

Protein was extracted from tissues or cells using RIPA lysis buffer supplemented with protease inhibitors. The protein samples were separated by SDS–polyacrylamide gel electrophoresis (SDS-PAGE) and then transferred onto polyvinylidene difluoride (PVDF) membranes. After blocking with 5% bovine serum albumin (BSA), the membranes were incubated overnight at 4 °C with primary antibodies against the following targets: PSRC1 (bs-7619R, Bioss), PCSK9 (ab185194, Abcam), MBD2 (sc-514062, Santa Cruz), TSG101 (ab320807, Abcam), CD63 (ab217345, Abcam), α-Tubulin (2146, Cell Signaling Technology), β-Actin (bs-0061R, Bioss), and GAPDH (bs-10900R, Bioss). Following extensive washes with TBST buffer, the membranes were incubated with horseradish peroxidase (HRP)-conjugated secondary antibodies at 37 °C for 1 h.

### Quantitative RT‒PCR (qRT‒PCR) assay

TRIzol reagent (Ambion, USA) was used to extract total RNA from cells or tissues. Then, the PrimeScript RT Reagent Kit (TaKaRa, Japan) was used for cDNA synthesis. The relative expression of genes was evaluated using PrimeScript RT‒PCR kits (TaKaRa, Japan) with GAPDH as the internal control. Primers were list in Supplementary Table S1.

### Analysis of atherosclerotic lesions

Following euthanasia, mice underwent systemic perfusion via the left ventricle. The entire aorta from the ascending aorta to the iliac bifurcation was then harvested, cleaned of adventitial fat, and opened longitudinally. The en face preparation was stained with Oil Red O and photographed on a blue background. For histological assessment, the aortic root was embedded in OCT compound for frozen sections (Oil Red O staining) or fixed in formalin for paraffin sections. Paraffin sections were stained with Hematoxylin and Eosin (H&E) to identify necrotic cores and with Masson’s trichrome to visualize collagen fibers. For immunofluorescence, frozen sections of the aortic root were stained with antibodies against CD68 (macrophages). Immunohistochemistry for α-SMA was performed on paraffin sections to identify vascular smooth muscle cells. All images were acquired using an Olympus microscope or a Leica confocal laser scanning microscope.

### EV isolation

EVs were isolated from macrophages-conditioned medium by differential ultracentrifugation. The medium was collected after 24 h of macrophages culture. The supernatant was sequentially centrifuged at 4 °C (300 × g for 10 min, 2000 × g for 10 min, and 10,000 × g for 30 min) to remove cells and large debris, followed by filtration through a 0.22 μm pore-size filter (Millipore). The clarified supernatant was then subjected to ultracentrifugation at 110,000 × g for 2 h at 4 °C using an Optima L-80 XP ultracentrifuge (Beckman Coulter, Brea, CA, USA). The resulting EV pellet was resuspended in PBS for immediate use or stored at − 80 °C.

### EV characterization

The size distribution and concentration of isolated EVs were analyzed using nanoparticle tracking analysis (NTA) with a NanoFCM instrument (N30E). EVs were appropriately diluted in filtered PBS and injected for measurement. Each sample was recorded three times and analyzed with NTA software. For morphological assessment, EVs were fixed with 2% glutaraldehyde, applied to a formvar/carbon-coated copper grid for 5 min at 25 °C, and negatively stained with 2% uranyl acetate for 1 min. After washing and drying, images were acquired using a Hitachi H7650 transmission electron microscope. The presence of EV-specific protein markers was confirmed by western blotting. Supernatant from both macrophages and isolated EVs were probed for TSG101 (ab320807, Abcam) and CD63 (ab217345, Abcam).

### Coculture of macrophage EVs with hepatocytes

Hepatocytes were seeded into 12-well plates at a density of 1 × 10^6 cells per well. After 24 h of culture, the medium was replaced with fresh culture medium. Subsequently, purified EVs (1 × 10^8 particles) isolated from the conditioned medium of macrophages were added to the hepatocyte medium. The co-culture was maintained at 37 °C for 24 h, after which the hepatocytes were collected for subsequent analysis.

### Inhibition of EV release

The release of EVs was pharmacologically inhibited using GW4869 in accordance with a previously established protocol. Briefly, macrophages were cultured until they reached approximately 80% confluence. The cells were then treated with 20 μM GW4869 (D1692, Sigma) diluted in complete DMEM containing 0.05% DMSO for 24 h. Subsequently, the medium was replaced with EV-depleted FBS medium supplemented with 20 μM GW4869, and the incubation continued for an additional 48 h. Following this treatment period, the conditioned medium was collected for subsequent EV isolation or co-culture experiments with recipient cells (hepatocytes). Control cells were treated in parallel with an equivalent volume of 0.05% DMSO solution, which served as the vehicle control.

### EV delivery in vivo

To evaluate the in vivo delivery of EVs carrying MBD2, macrophages were transfected with an MBD2-overexpressing adenovirus and then cultured. The secreted EVs (EVs-MBD2) were collected and purified. Eight-week-old mice on a HFD received intravenous injections of these EVs via the tail vein at a dose of 1 × 10^^10^ particles per animal every three days. After the treatment period, vascular tissue samples were collected for subsequent analysis.

### Co-immunoprecipitation

For co-immunoprecipitation (Co-IP) assays, cells were treated as indicated and harvested at 60%–70% confluence. Total protein was extracted using RIPA lysis buffer containing protease inhibitors. Equal amounts of protein lysate were incubated overnight at 4 °C with rotation, using an anti-PSRC1 antibody or a species-matched normal IgG as a negative control. Subsequently, protein A/G magnetic beads were added to each sample to capture the antibody-protein complexes and incubated for an additional 2 h at 4 °C. The beads were then washed extensively with ice-cold lysis buffer to remove non-specifically bound proteins. The immunoprecipitated complexes were eluted from the beads by boiling in 1 × SDS-PAGE loading buffer at 95 °C for 10 min. The eluted proteins were then separated by SDS-PAGE and analyzed by Western blotting to detect co-precipitated target proteins.

### ChIP analysis and transcriptional activation activity

Chromatin immunoprecipitation (ChIP) assays were performed using an anti-MBD2 antibody according to the manufacturer’s instructions for the ChIP assay kit (Millipore, USA). Briefly, approximately 1 × 10^7 hepatocytes were cross-linked with 1% formaldehyde for 10 min at room temperature. Chromatin was then sheared by sonication to yield DNA fragments of 200–500 bp. Following pre-clearing, 5–10 µg of sheared chromatin was incubated overnight at 4 °C with rotation using a specific anti-MBD2 antibody. Normal rabbit IgG served as a negative control. Antibody-protein-DNA complexes were captured with Protein A/G agarose beads, washed sequentially, and eluted. After cross-link reversal and DNA purification, the enrichment of MBD2 at specific PCSK9 promoter regions was quantified by qPCR using SYBR Green. Primer sequences were designed to flank predicted CpG-rich regions within the PCSK9 promoter.

To predict CpG islands and design primers, the sequence of the PCSK9 promoter region (− 2000 to 0) was analyzed using the online tool MethPrimer Promoter 2.0 (http://www.urogene.org/cgi-bin/methprimer2/MethPrimer.cgi). Precipitated DNA was amplified by PCR with the primers targeting CpG-rich regions within the PCSK9 promoter (Supplementary Table S1).

To evaluate the transcriptional activation activity of PCSK9, a dual-luciferase reporter assay was conducted using the Promega kit according to established protocols.

### Adenoviral transduction and treatment (in vitro)

For in vitro overexpression, a recombinant adenovirus encoding the mouse Psrc1 gene (Ad-PSRC1) and an empty control vector (Ad-NC) were synthesized by Genechem. Macrophages were seeded in 6-well plates and reached 70% confluence before transduction. Cells were infected with Ad-PSRC1 or Ad-NC at a multiplicity of infection (MOI) of 50 in serum-free medium for 6 h. The medium was then replaced with complete DMEM containing 10% FBS. Functional assays and molecular analyses were performed 48 h post-transduction. Overexpression efficiency was validated via qRT-PCR and Western blot.

### Adenoviral injection for gene knockdown (in vivo)

To knockdown MBD2 in vivo, an adenoviral vector expressing short hairpin RNA (shRNA) against Mbd2 (Ad-shMBD2) and a corresponding control (Ad-shNC) were used. The shRNA sequence targeting Mbd2 was 5′- CGGCTCCATCAGCAAGTTTAA − 3′. Mice received a single tail vein injection of the adenovirus at a dose of 1*10^^9^ PFU in 100 uL of sterile PBS. Animals were sacrificed 7 days post-injection, and liver tissues were harvested to confirm knockdown efficiency and perform subsequent analyses.

### Macrophage-specific knockdown via AAV6 (in vivo)

For macrophage-specific knockdown of Psrc1 in vivo, an adeno-associated virus serotype 6 (AAV6) vector was constructed. The expression of shRNA targeting Psrc1 was driven by the macrophage-specific F4/80 promoter (AAV6-F4/80-shPsrc1). Mice were systemically administered with AAV6-F4/80-shPsrc1 or the control vector (AAV6-F4/80-shNC) at a dose of 1*10^^11^ vg per mouse via tail vein injection. To ensure stable and specific gene silencing, experimental procedures were initiated 3 weeks post-injection. The specificity and efficiency of the macrophage-targeted knockdown were verified by isolating primary macrophages from the mice and assessing Psrc1 levels.

### Statistical analysis

Statistical analyses were performed using GraphPad Prism (version 9.0). Data are expressed as the mean ± standard deviation (S.D.). Differences between two groups were evaluated using an unpaired two-tailed Student’s t test. For comparisons among more than two groups, one-way analysis of variance (ANOVA) was applied, followed by Bonferroni’s post hoc test. A P value of less than 0.05 was considered statistically significant.

## Supplementary Information

Supplementary Material 1

Supplementary Material 2

Supplementary Material 3

## Data Availability

All data generated or analyzed during this study are included in this article and its supplementary information files.

## Abbreviations

ApoE^−/−^: Apolipoprotein E–deficient
ASCVD: Atherosclerotic cardiovascular disease
ChIP: Chromatin immunoprecipitation
Co-IP: Co-immunoprecipitation
EVs: Extracellular vesicles
H&E: Hematoxylin and eosin
HDL-C: High-density lipoprotein cholesterol
MBD2: Methyl-CpG–binding domain protein 2
NTA: Nanoparticle tracking analysis
PCSK9: PROPROTEIN convertase subtilisin/kexin type 9
PSRC1: Proline/serine-rich coiled-coil protein 1
qRT-PCR: Quantitative real-time polymerase chain reaction
SMC: Smooth muscle cell
TEM: Transmission electron microscopy
TSG101: Tumor susceptibility gene 101
