# Supplementary material for: Macrophage PSRC1 attenuates atherosclerosis via extracellular vesicle-mediated MBD2 delivery to induce PCSK9 promoter methylation in hepatocytes
Source: Cell Biosci. 2026 Jun 9;16:92. doi: 10.1186/s13578-026-01598-9 (PMC13479547; doi:10.1186/s13578-026-01598-9)
Supplement: Supplementary file 1 [file 13578_2026_1598_MOESM1_ESM.pdf]

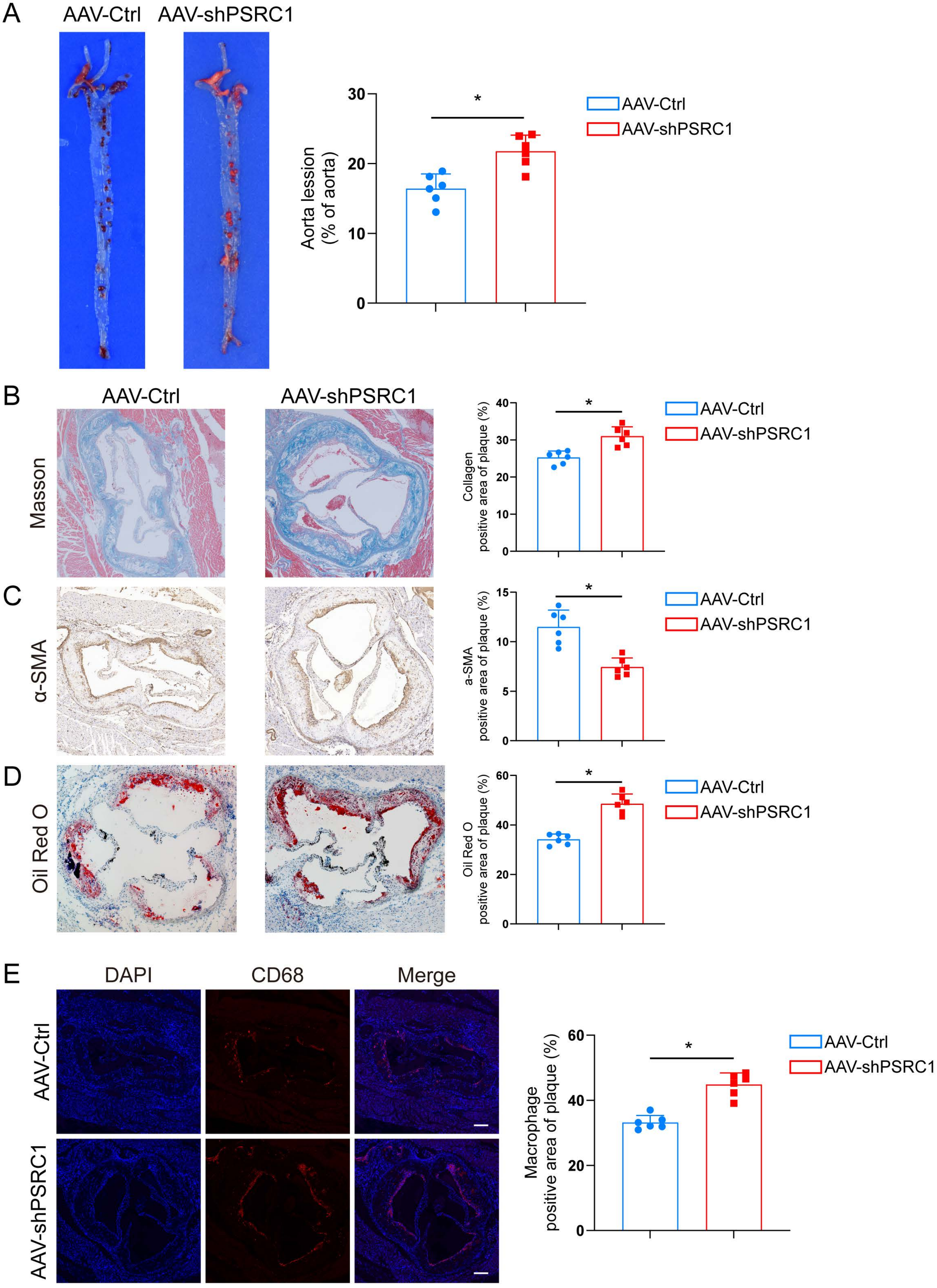

A
AAV-Ctrl
AAV-shPSRC1
Aorta lession (% of aorta)
30
20
10
0
*
AAV-Ctrl
AAV-shPSRC1
B
AAV-Ctrl
AAV-shPSRC1
Masson
Collagen positive area of plaque (%)
40
30
20
10
0
*
AAV-Ctrl
AAV-shPSRC1
C
α-SMA
a-SMA positive area of plaque (%)
15
10
5
0
*
AAV-Ctrl
AAV-shPSRC1
D
Oil Red O
Oil Red O positive area of plaque (%)
60
40
20
0
*
AAV-Ctrl
AAV-shPSRC1
E
DAPI
CD68
Merge
AAV-Ctrl
AAV-shPSRC1
Macrophage positive area of plaque (%)
60
40
20
0
*
AAV-Ctrl
AAV-shPSRC1

Figure S1

A

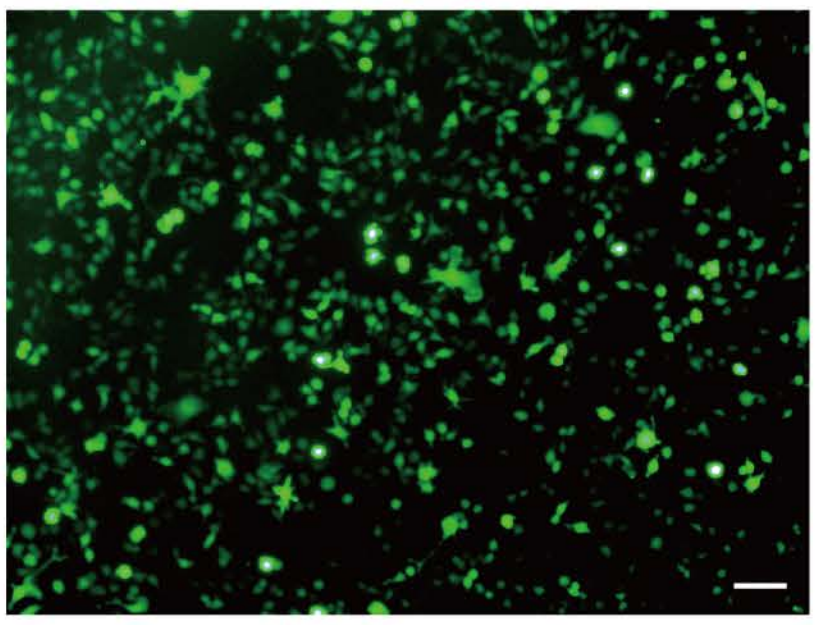

Figure S2

A

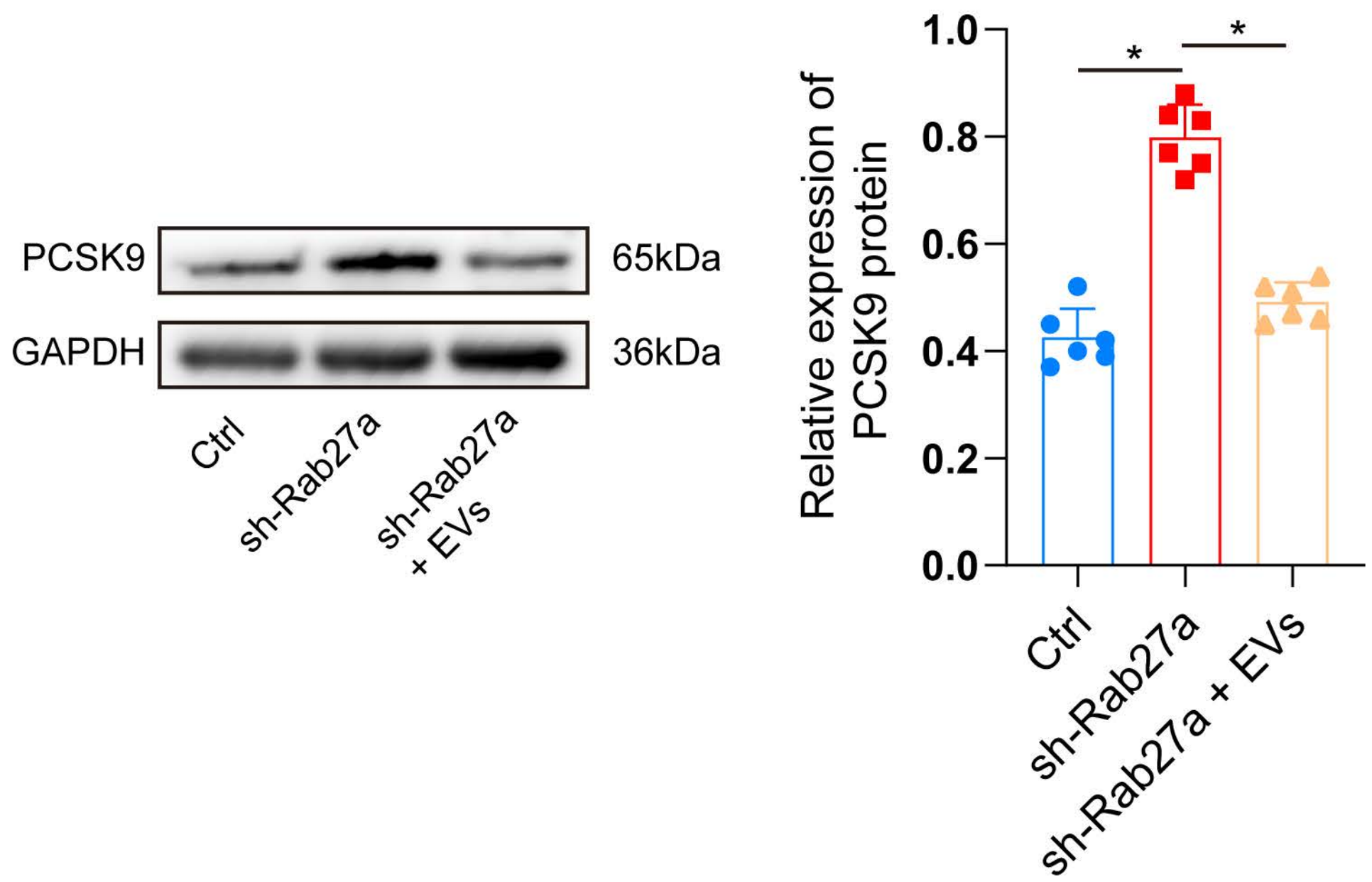

PCSK9
GAPDH
65kDa
36kDa
Ctrl
sh-Rab27a
sh-Rab27a + EVs
Relative expression of PCSK9 protein
1.0
0.8
0.6
0.4
0.2
0.0
*
*
Ctrl
sh-Rab27a
sh-Rab27a + EVs

Figure S3

A

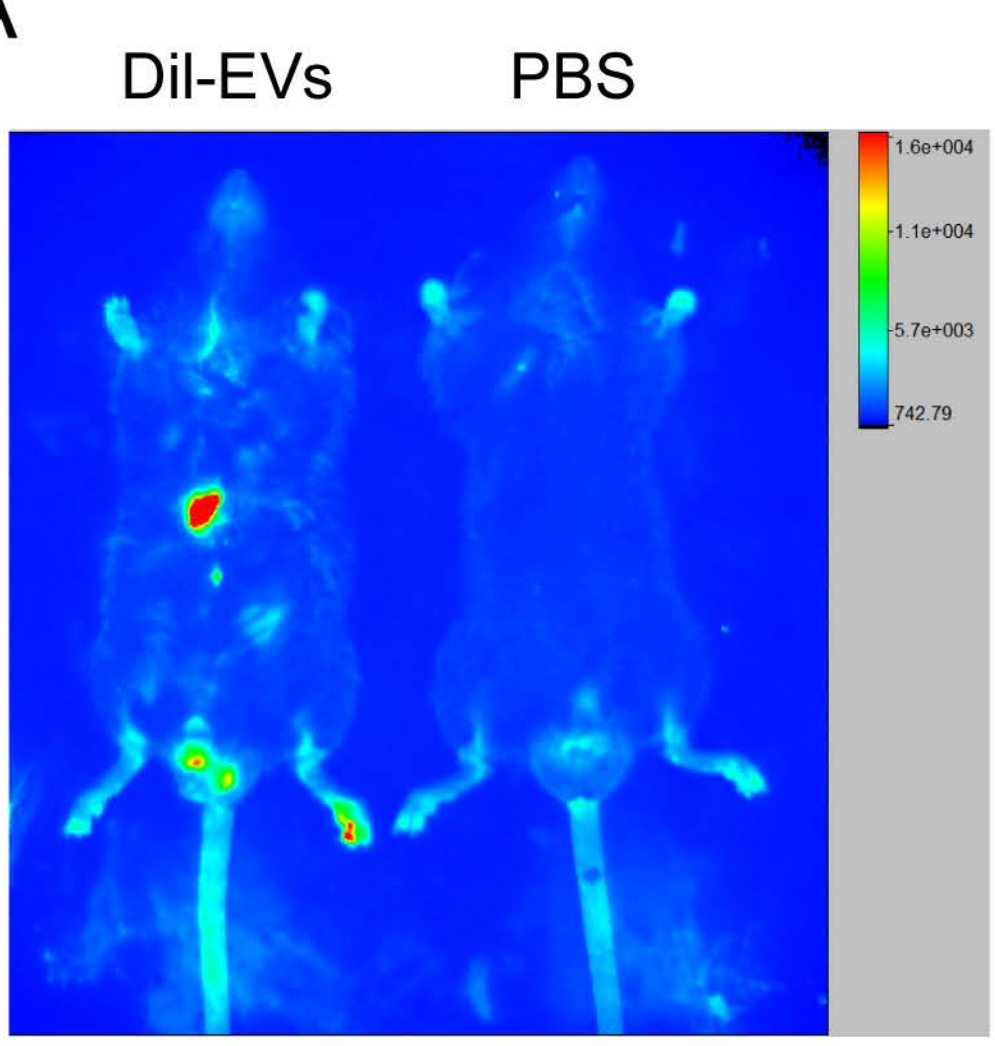
Dil-EVs
PBS
1.6e+004
1.1e+004
5.7e+003
742.79

B

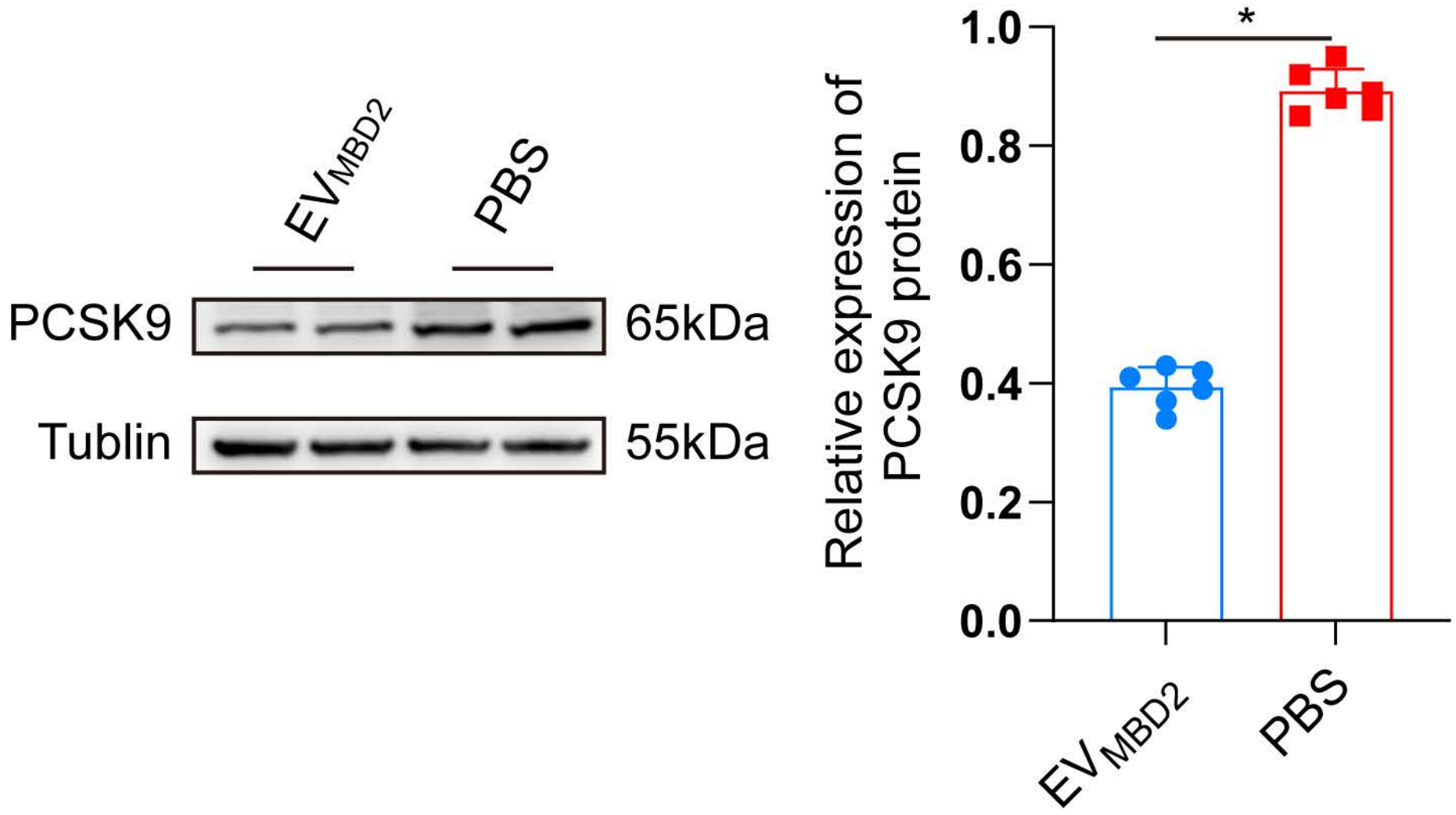
EVMBD2
PBS
PCSK9
65kDa
Tublin
55kDa
Relative expression of PCSK9 protein
1.0
0.8
0.6
0.4
0.2
0.0
*
EVMBD2
PBS

Figure S4

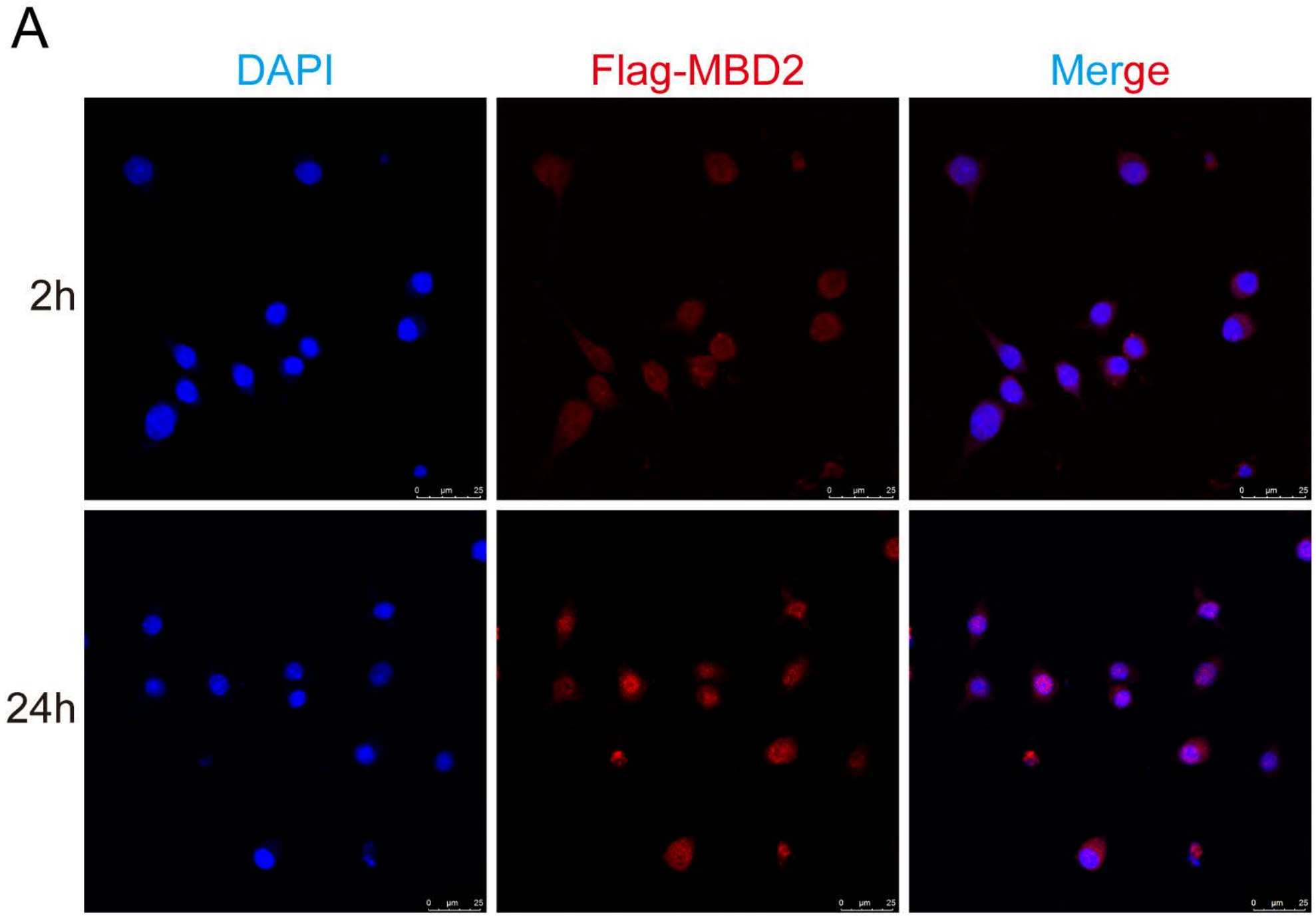

Figure S5

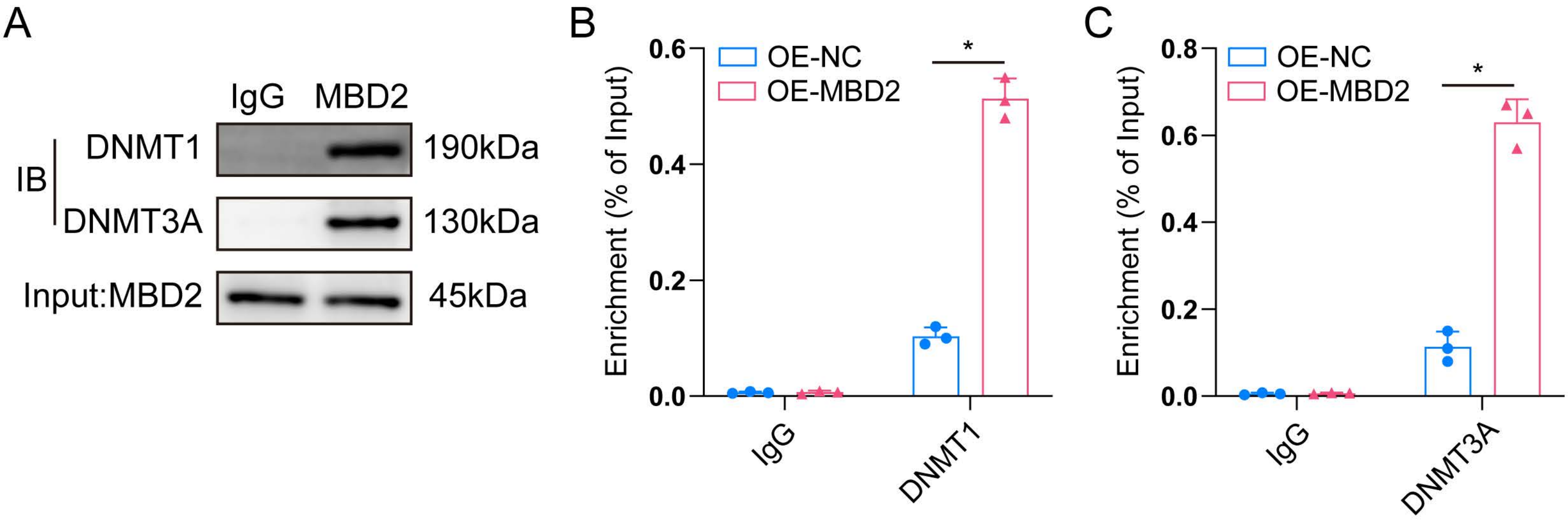
A
IgG
MBD2
IB
DNMT1
190kDa
DNMT3A
130kDa
Input:MBD2
45kDa
B
OE-NC
OE-MBD2
*
Enrichment (% of Input)
0.0
0.2
0.4
0.6
IgG
DNMT1
C
OE-NC
OE-MBD2
*
Enrichment (% of Input)
0.0
0.2
0.4
0.6
0.8
IgG
DNMT3A

Figure S6
