# Supplementary material for: Macrophage PSRC1 attenuates atherosclerosis via extracellular vesicle-mediated MBD2 delivery to induce PCSK9 promoter methylation in hepatocytes
Source: Cell Biosci. 2026 Jun 9;16:92. doi: 10.1186/s13578-026-01598-9 (PMC13479547; doi:10.1186/s13578-026-01598-9)
Supplement: Supplementary file 2 [file 13578_2026_1598_MOESM2_ESM.docx]

**Supplementary Figure Legend**

**Fig. S1. Macrophage-specific knockdown of PSRC1 exacerbates atherosclerotic lesion progression and instability.**

To validate the cell-intrinsic role of macrophage PSRC1, mice were treated with AAV6-F4/80-shPsrc1 or AAV-NC. (A) Representative images and quantification of Oil Red O-stained whole aortas, showing overall plaque burden. (B) Masson’s trichrome staining of aortic root sections to evaluate collagen content and fibrotic areas. (C) Immunohistochemical staining for α-SMA in aortic roots, indicating the distribution of smooth muscle cells within the lesions. (D) Representative images and quantification of Oil Red O-stained aortic root cross-sections, highlighting the lipid core size. (E) Immunofluorescence (IF) staining for CD68 and DAPI in aortic roots to assess macrophage infiltration.

**Fig. S2. Validation of PSRC1 transduction efficiency in macrophages.**

(A) Representative fluorescence images of macrophages at 48 h post-transduction with either OE-NC or OE-PSRC1 adenovirus (expressing GFP).

**Figure S3. Macrophage-mediated suppression of hepatic PCSK9 is dependent on the EV secretory pathway.**

Representative Western blot and quantitative analysis of PCSK9 protein levels in hepatocytes. Macrophages were subjected to the following treatments: (1) Control: hepatocytes incubated with the secretome of control macrophages; (2) sh-Rab27a: hepatocytes incubated with the secretome of Rab27a-silenced macrophages (blocking EV release); (3) sh-Rab27a + EVs: hepatocytes incubated with the Rab27a-silenced secretome supplemented with isolated, exogenous macrophage-derived EVs. GAPDH was used as the loading control for hepatocytes. *P < 0.05.

**Fig. S4. Hepatic targeting and functional validation of administered EVs in vivo.**

(A) Representative live animal fluorescence imaging of mice 12 h after tail vein injection of DiI-labeled EV-MBD2 or PBS. The pseudo-color heat map indicates fluorescence intensity, demonstrating preferential enrichment of EVs in the liver. (B) Representative Western blot and quantitative analysis of hepatic PCSK9 protein levels in mice treated with PBS or EV-MBD2. *P < 0.05

**Figure S5. EV-derived MBD2 translocates to the nucleus of recipient hepatocytes in a time-dependent manner.**

Representative confocal immunofluorescence images of hepatocytes incubated with EVs derived from Flag-MBD2-overexpressing macrophages. Cells were fixed at 2 h and 24 h post-incubation. Exogenous MBD2 was visualized using an anti-Flag antibody (green), and nuclei were counterstained with DAPI (blue). At 2 h, the signal was primarily cytoplasmic, while by 24 h, significant nuclear accumulation was observed.

**Fig. S6. MBD2 recruits DNMT1 and DNMT3A to catalyze PCSK9 promoter methylation.**

(A) Co-immunoprecipitation (Co-IP) analysis of the interaction between MBD2 and DNMTs. MBD2 was immunoprecipitated from hepatocyte lysates, and the co-precipitated DNMT1 and DNMT3A were detected by Western blot. IgG served as a negative control.

(B-C) ChIP-qPCR analysis of DNMT1 (B) and DNMT3A (C) enrichment at the PCSK9 promoter in hepatocytes transfected with OE-NC or OE-MBD2. Chromatin was immunoprecipitated with anti-DNMT1, anti-DNMT3A, or control IgG antibodies. Enrichment levels are presented as a percentage of the total input DNA (% of input). *P < 0.05.
