# Supplementary material for: Macrophage PSRC1 attenuates atherosclerosis via extracellular vesicle-mediated MBD2 delivery to induce PCSK9 promoter methylation in hepatocytes
Source: Cell Biosci. 2026 Jun 9;16:92. doi: 10.1186/s13578-026-01598-9 (PMC13479547; doi:10.1186/s13578-026-01598-9)
Supplement: Supplementary file 3 [file 13578_2026_1598_MOESM3_ESM.doc]

Supplementary Table S1: The primers used in this study.

| Primers name |  | Sequence (5’-3’) |
| --- | --- | --- |
| PSRC1 | + | TCAAGTTCATTGTGGACGAGAC |
|  | - | GGGAGCTACTTCATTTGGGTT |
| PCSK9 | + | AGCAGCCAGGTGGAGGTGTATC |
|  | - | GCCTGTCTGTGGAAGCGTGTC |
| GAPDH | + | TGACCTCAACTACATGGTCTACA |
|  | - | CTTCCCATTCTCGGCCTTG |
| PCSK9 promoter F1 | + | GAGAGGAGGAGTTTTTAGGG |
| PCSK9 promoter R1 | - | AAAACCTTCTAAAATATAAATACTTAAC |
| PCSK9 promoter F2 | + | GAGAGGAGGAGTTTTTAGGG |
| PCSK9 promoter R2 | - | AAACCTTCTAAAATATAAATACTTAAC |
| PCSK9 promoter F3 | + | GGAGAGGAGGAGTTTTTAGGG |
| PCSK9 promoter R3 | - | AAAACCTTCTAAAATATAAATACTTAAC |
| PCSK9 promoter F4 | + | AAAACCTTCTAAAATATAAATACTTAAC |
| PCSK9 promoter R4 | - | AGGAAGATCGGGCATA |
| PCSK9 promoter F5 | + | GGAGAGGAGGAGTTTTTAGGG |
| PCSK9 promoter R5 | - | AAACCTTCTAAAATATAAATACTTAAC |
